# Magnetic anisotropy in Shiba bound states across a quantum phase transition

**DOI:** 10.1038/ncomms9988

**Published:** 2015-11-25

**Authors:** Nino Hatter, Benjamin W. Heinrich, Michael Ruby, Jose I. Pascual, Katharina J. Franke

**Affiliations:** 1 Fachbereich Physik, Freie Universität Berlin, Arnimallee 14, 14195 Berlin, Germany; 2 CIC nanoGUNE and Ikerbasque, Basque Foundation for Science, Tolosa Hiribidea 78, Donostia-San Sebastian 20018, Spain

## Abstract

The exchange coupling between magnetic adsorbates and a superconducting substrate leads to Shiba states inside the superconducting energy gap and a Kondo resonance outside the gap. The exchange coupling strength determines whether the quantum many-body ground state is a Kondo singlet or a singlet of the paired superconducting quasiparticles. Here we use scanning tunnelling spectroscopy to identify the different quantum ground states of manganese phthalocyanine on Pb(111). We observe Shiba states, which are split into triplets by magnetocrystalline anisotropy. Their characteristic spectral weight yields an unambiguous proof of the nature of the quantum ground state. Our results provide experimental insights into the phase diagram of a magnetic impurity on a superconducting host and shine light on the effects induced by magnetic anisotropy on many-body interactions.

## Abstract (web summary)

The exchange coupling strength between magnetic adsorbates and a superconducting surface determines the nature of the system's quantum ground state. Here, the authors use scanning tunnelling microscopy to explore the ground state and excited state properties of manganese phthalocyanine adsorbed on a Pb(111) surface.

## Graphical abstract

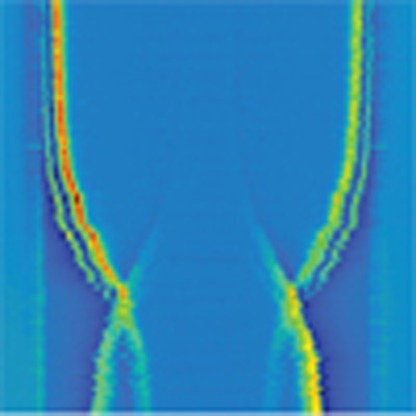

Magnetic adsorbates on a superconductor create a magnetic scattering potential for the quasi-particles of the superconductor. A single spin gives rise to so-called Yu–Shiba–Rusinov (Shiba) states^1^,^2^,^3^. In recent times, it was argued that hybridization of the Shiba states can lead to Shiba bands with nontrivial topological character^4^,^5^,^6^. This is essential for the formation of Majorana modes, which have been detected in ferromagnetic chains of Fe atoms on a Pb(110) surface^5^. If not only one but several Shiba states are present, the hybridization will lead to a more complex band structure. Different origins of multiple Shiba states are discussed theoretically. These include different angular momentum scattering contributions, individual *d* orbitals acting as separate scattering potentials or low-energy excitations due to magnetic anisotropy or vibrations^7^,^8^,^9^,^10^,^11^,^12^. However, experimentally, the origin of multiple Shiba states is often difficult to determine^13^,^14^.

Concomitantly with the formation of a Shiba state, the exchange coupling between the magnetic adsorbate and the substrate also drives the formation of a singlet Kondo state^10^,^15^,^16^,^17^. If the Kondo energy scale *k*_B_*T*_K_ (where *k*_B_ is the Boltzmann factor and *T*_K_ is the Kondo temperature) is much larger than the superconducting pairing energy Δ, the Kondo-screened state is the ground state. On the other hand, if 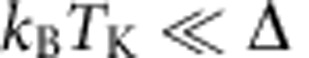, an unscreened free-spin state with a spin *S*>0 is the ground state. A Shiba resonance as fingerprint of this magnetic interaction can be found in the quasiparticle excitation spectra within the superconducting energy gap if *k*_B_*T*_K_∼Δ (ref. 18). This resonance corresponds to a transient state, in which an electron is added/removed to/from the ground state. Thus, the electron occupation changes and the spin is altered by Δ*S*=±1/2. In the Kondo-screened case, the Shiba resonance is found with a binding energy *E*_b_ below the Fermi level *E*_F_. The weaker the exchange coupling and Kondo screening, the closer is the Shiba state to the Fermi level. The crossing of the Shiba state through *E*_F_ marks the quantum phase transition from the Kondo-screened *S*=0 to the free-spin state on the superconductor, that is, *S*>0. This transition occurs at *k*_B_*T*_K_∼0.3Δ and has been described theoretically^15^,^17^,^19^ and experimentally^16^,^20^,^21^.

Spin-1/2 systems feature one pair of Shiba resonances at ±*E*_b_ (ref. 18). If the adsorbate carries a higher spin, multiple Shiba states may appear inside the gap as discussed theoretically by Žitko *et al.*^10^ They argue that such systems may involve multiple Kondo screening channels with different coupling strengths *J*_*k*_ and, hence, multiple Shiba states. Furthermore, a mutual coupling of the spins may lead to a splitting of the peaks in the excitation spectra in the presence of magnetic anisotropy. Here we use scanning tunnelling microscopy (STM) and spectroscopy to resolve triplets of Shiba states on single paramagnetic molecules. A multitude of different adsorption sites provides access to a large range of magnetic coupling strengths with the substrate. We detect the splitting of the Shiba states even throughout the quantum phase transition from a Kondo-screened to a free-spin ground state. The intensities of the Shiba resonances yield an unambiguous proof of the nature of the spin states and their splitting by magnetic anisotropy. The basic understanding of the influence of magnetic anisotropy on many-body interactions is crucial for the design of quantum states with controlled properties. Furthermore, its knowledge may provide interesting approaches for creating and addressing Majorana states in proximity-coupled magnetic nanostructures^12^.

## Results

### Detection of Shiba states

The effects of magnetic anisotropy on Shiba states can be explored experimentally by bringing a metal-organic molecule into contact with a superconductor. The organic ligand is responsible for the splitting of the spin states with *S*≥1 of the transition metal core^22^,^23^. Manganese phthalocyanine (MnPc) has a spin *S*=3/2 in gas phase and retains a magnetic moment on metal surfaces^24^,^25^,^26^. In particular, its magnetic moment interacts with the superconductor Pb and shows single Shiba states when measured at 4.5 K^16^.

**Figure 1:**
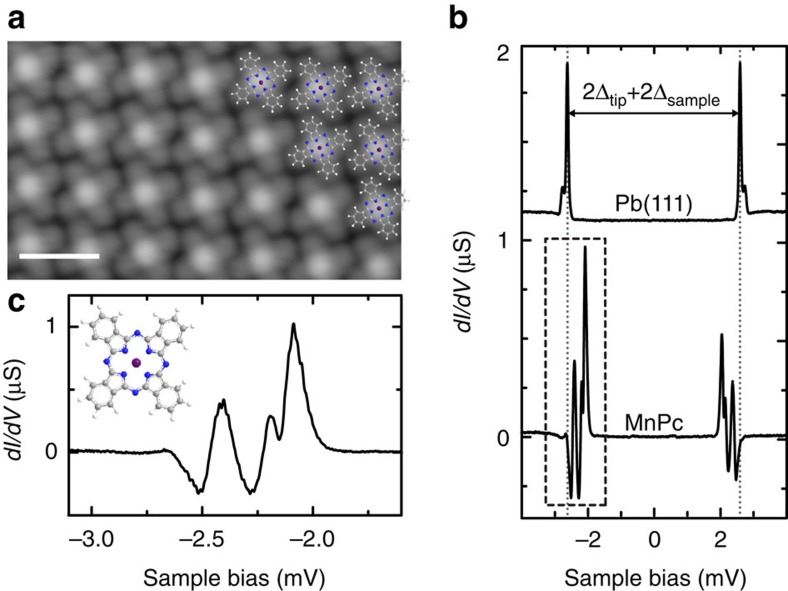
MnPc adsorption and magnetic fingerprint on Pb(111). (**a**) STM topography of a MnPc monolayer island on Pb(111), partially overlaid with ball-and-stick structure models of MnPc (*V*=50 mV, *I*=200 pA); scale bar is 2 nm. (**b**) *dI*/*dV* spectra on the pristine Pb(111) surface (offset for clarity) and on the centre of an MnPc molecule inside a molecular island. Grey dotted lines indicate the energy of the BCS quasiparticle resonances (opening feedback loop at: *V*=5 mV, *I*=200 pA). (**c**) Magnified view on the subgap excitations below *E*_F_ in the dashed box of the MnPc spectrum in **b**. The inset shows an enlarged MnPc structure model.

Deposition of MnPc molecules at room temperature results in self-assembled, densely packed monolayer islands (see Fig. 1a). In topography, the molecules appear clover shaped with four lobes around a central protrusion of the Mn ion. The nearly square lattice of the molecular adlayer accommodates many different adsorption sites of the Mn core on the hexagonal Pb lattice. Consequently, this Moiré-like pattern involves variations in the electronic and magnetic coupling strength between adsorbate and substrate^16^,^25^. This rich system allows us to identify different quantum ground states with distinct fingerprints of their magnetic excitations.

We use tunnelling spectroscopy with a superconducting Pb tip at 1.2 K, to detect fingerprints of magnetic interaction of MnPc with the superconducting substrate. As a reference, we plot the differential conductance (*dI*/*dV*) spectrum of the bare Pb surface in Fig. 1b. A region of zero conductance around *E*_F_, that is, the superconducting gap, is framed by quasiparticle resonances at *eV*=±(Δ_sample_+Δ_tip_)=±2.63 meV, with Δ_sample_ and Δ_tip_ being the pairing energy of the sample and the tip, respectively. The observed presence of the two quasiparticle resonances at each side of the gap is explained by the two-band superconductivity of the Pb single crystal as described recently^27^.

Interestingly, the spectra on the MnPc molecules show two triplet sets of peaks inside the superconducting energy gap, which are symmetric in energy around *E*_F_, but asymmetric in intensity (Fig. 1b). In the limit of small tunnelling rates—as in our experiment—the asymmetric intensity is an expression of the different hole and electron components of the Shiba wavefunctions^14^. The different weights arise from the particle-hole asymmetry in the normal state and an on-site Coulomb potential at the scattering site^9^,^17^,^28^.

The triplets consist of very sharp peaks (50–100 μeV full width at half maximum), which are separated by up to 400 μeV (for example, see Fig. 1c). To observe such narrow peaks at 1.2 K, a superconducting tip is required, because then the resolution is not limited by the Fermi–Dirac distribution anymore^27^. Yet, finite temperatures lead to finite lifetime effects, which broaden the superconducting coherence peaks and subgap states. Hence, already at 4.5 K, the triplet peaks overlap and give rise to a single peak^16^.

**Figure 2:**
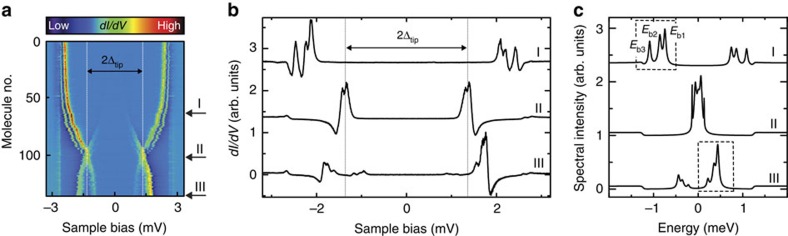
Differential conductance spectra of MnPc in Moiré-like pattern. (**a**) False colour plot of *dI*/*dV* spectra of 137 MnPc molecules ordered by the energy of the most intense Shiba resonance (feedback: *V*=5 mV, *I*=200 pA). The colour bar indicates the intensity of the *dI*/*dV* traces in the plot. (**b**) Spectra of three MnPc molecules with bound-state energies in three different coupling regimes. (**c**) Spectral intensity obtained by deconvolution of the spectra shown in **b**. The bound state resonances are labelled *E*_b1_, *E*_b2_ and *E*_b3_, respectively. Dashed boxes highlight the parts in the spectra relevant for the quantitative analysis of the peak areas and splittings.

The varying coupling strength within the Moiré-like structure leads to different bound-state energies^16^. We use this property to further investigate the origin of the splitting of the Shiba resonances and perform tunnelling spectroscopy on more than 130 molecules. All spectra exhibit two triplets of peaks: one in the bias voltage window −2Δ/*e*<*V*_bias_<−Δ/*e* and one in Δ/*e*<*V*_bias_<2Δ/*e*. The additional resonances in the bias interval [−Δ_tip_/*e*, Δ_tip_/*e*] are due to tunnelling into/out of thermally excited Shiba states (Supplementary Fig. 1 and Supplementary Note 1). In Fig. 2a, we ordered the spectra according to the energy of the most intense Shiba resonance. The false colour plot shows a collective shift of the Shiba triplets through the superconducting gap. The spectra can be categorized into three different regimes. For each of these, we plot a spectrum in Fig. 2b. In spectrum I, the intensity of the triplet is larger for tunnelling out of the occupied states; in spectrum II, the peaks are close to ±Δ_tip_, which hinders a clear distinction of the triplet; spectrum III exhibits larger intensity of the triplet when tunnelling into unoccupied states. The energetic position of the higher-intensity subgap peaks corresponds to the binding energy *E*_b_ of the Shiba states^15^,^16^,^17^. The order of the spectra from top to bottom thus represents a decreasing coupling strength *J* with the substrate, which comes along with different adsorption sites. The three spectra are representative for the Kondo-screened case (*E*_b_<0, spectrum I in Fig. 2b), the free-spin case (*E*_b_>0, spectrum III) and a case close to the quantum phase transition (*E*_b_≈0, spectrum II).

### Possible origins of a Shiba state splitting

The collective shift of the Shiba states through a wide range of the gap (and even the quantum phase transition) suggests a correlated origin of the peaks within the triplet. In principle, three different scenarios may account for the occurrence of multiple Shiba bound states in a type I superconductor: different angular momentum scattering channels^7^,^8^,^9^,^12^,^13^, independent scattering at spins in different *d* orbitals^10^ and bound-state excitations coupled to other low-energy excitations, such as spin excitations or vibrations^10^,^11^.

Bound states, which originate from higher angular momentum scattering channels (*l*=1, 2 and so on), always reside close to the gap edge, whereas the *l*=0 channel may shift through the superconducting energy gap depending on the coupling strength *J*^9^,^12^. This is in contrast to our observation and hence we can rule out different angular momentum scattering channels as possible origin for the split bound states. In the case of independent scattering of spins in different *d* orbitals, a similar shift of all *E*_b*i*_ is unexpected, because *d* orbitals exhibit different symmetries and interactions with the surface.

For the coupling to other degrees of freedom at a similar energy scale, a collective shift is expected. As we will show in the following, a detailed analysis of the intensities of the Shiba states allows us to unambiguously identify a magnetocrystalline origin of the splitting of the Shiba states as predicted by Žitko *et al.*^10^ for *S*≥1 systems.

### Shiba intensity as fingerprint of a quantum phase transition

The evaluation of the Shiba intensities also sustains the assigned regimes of Kondo-screened and free-spin ground states. Such an analysis requires the spectral density of the molecule–substrate system, which can be directly related to the relative weight in the tunnelling processes. For this, we remove the effect of the superconducting density of states of the tip by numerical deconvolution of the spectra (Fig. 2c) as described in the Methods section (see Supplementary Fig. 2 for fit quality).

**Figure 3:**
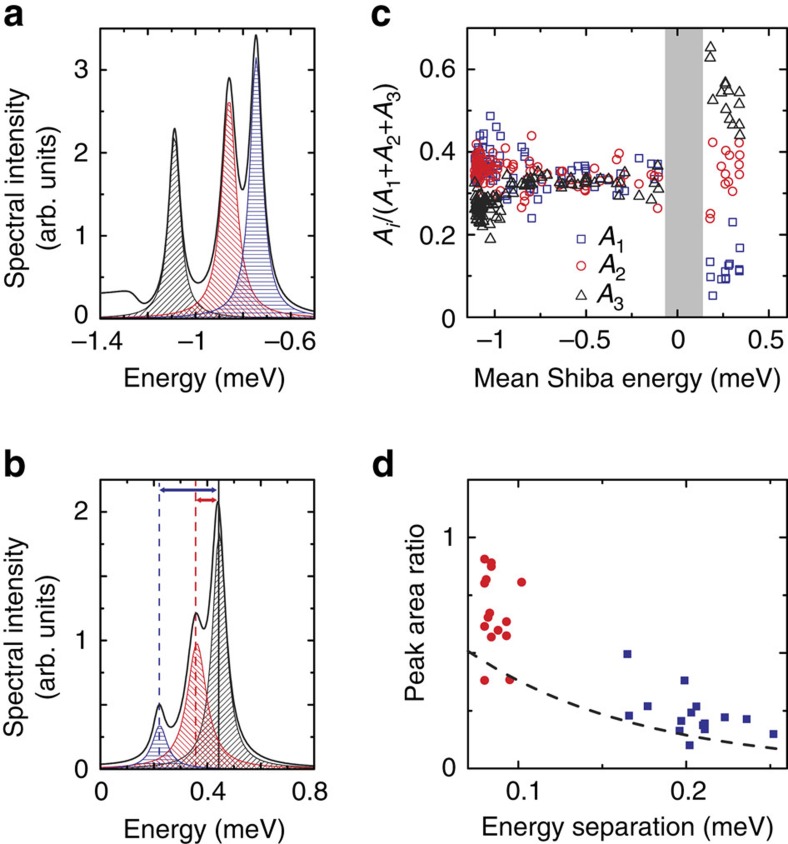
Shiba state analysis. (**a**) Zoom on the relevant part of the deconvoluted spectrum I (Kondo screened). We model the spectrum with three Lorentzian peaks of different width (shaded in blue, red and grey) and a broadened step function at the gap edge. (**b**) As in **a**, but on spectrum III (free spin). (**c**) Relative peak areas 
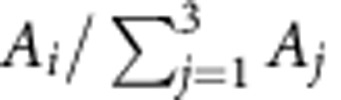
 of the Shiba bound states plotted versus the mean energy 
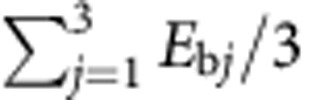
 of the respective Shiba triplet. The grey shaded area indicates the region close to the quantum phase transition, where the Shiba resonances significantly overlap with their thermally excited counterparts. This prohibits a clear distinction of the peak areas. The data are therefore omitted in the plot. (**d**) Ratio of peak areas in the free-spin regime. *A*_1_/*A*_3_ (blue squares) and *A*_2_/*A*_3_ (red circles) are plotted versus the energy separation (as sketched in **b** by blue and red arrows, respectively). A Boltzmann distribution for *T*=1.2 K is sketched as dashed line.

We observe a distinct change in the relative peak areas within the triplets when crossing the quantum phase transition. In the Kondo-screened regime, that is, for Shiba states with negative binding energies, the individual peaks within a triplet exhibit equal areas (Fig. 3a). Thus, their relative areas 
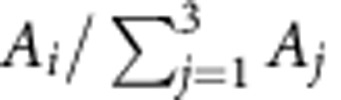
 are close to 1/3 (data points at negative Shiba-binding energies in Fig. 3c). In contrast, in the free-spin regime, that is, positive Shiba-binding energies, the areas are considerably different (Fig. 3b,c). Here, the relative area of the subgap excitations decreases from the outermost, *E*_b3_, to the innermost, *E*_b1_. The ratio of peak areas decreases with increasing energy separation (Fig. 3d). Such a behaviour is reminiscent of a Boltzmann distribution, indicating a thermal occupation of a split many-body ground state.

The characteristic change in the relative peak areas at the point of the quantum phase transition is a direct fingerprint of the origin of the splitting. Independent bound states of different *d* orbitals would not change their relative weight at the phase transition. Furthermore, the phase transition should not occur simultaneously for all scattering channels. Additional vibronic resonances, that is, the coupling to other degrees of freedom, would appear as satellite peaks at higher absolute energy for both ground states. Their spectral weight should scale with the electron–phonon coupling strength, albeit being substantially lower than the weight of the main resonance^11^. This is clearly not the case in our data. Hence, both scenarios can safely be ruled out.

### Scattering channels and anisotropy splitting of Shiba states

**Figure 4:**
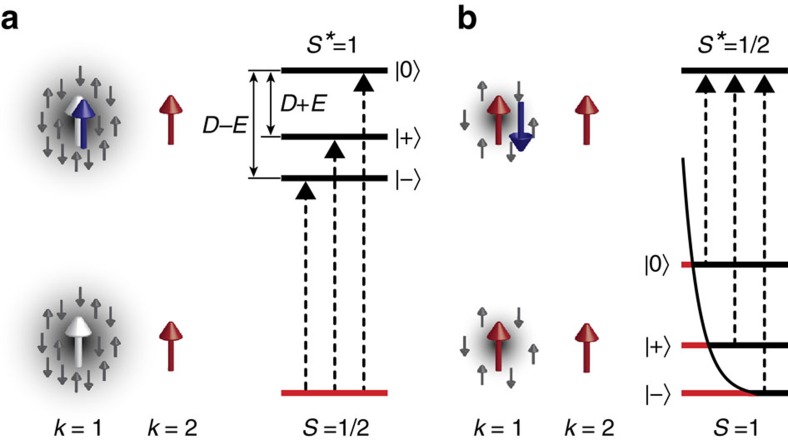
Illustration of the many-body states and the related energy level diagrams. (**a**) In the Kondo-screened ground state, the spin (white arrow) in scattering channel *k*=1 is screened, illustrated by grey arrows and shading, and the tunnelling electron can enter with its spin (blue arrow) parallel to the spin in *k*=2, increasing the excited state's total spin to S*=1. The excitation scheme including the anisotropy-split excited state is shown by the energy-level diagram with indicated anisotropy parameters *D* and *E*. (**b**) In the free-spin ground state, the spin in *k*=1 (red arrow) is only partially screened. The tunnelling electron has to enter this state in an anti-parallel alignment, obeying the Pauli exclusion principle and reducing the spin in the excited state to S*=1/2. Red lines in the excitation scheme symbolize the thermal occupation of the anisotropy-split ground state as determined by the Boltzmann distribution function (black line).

To conclude on the correct model for the description of the multiple subgap resonances, we summarize the essential properties of the Shiba states: On the one hand, the equal peak areas for the Kondo-screened ground state reflect that our system is characterized by a single level with three possible excitation levels of equal probability. On the other hand, the Boltzmann-like distribution of the areas in the free-spin case indicate a triplet-split ground state with one excited state. We can correlate these levels to the magnetic interaction channels of the MnPc molecule with the substrate. The MnPc molecule carries a spin *S*=3/2 in gas phase^29^. Theory predicts that on Pb(111), the spin in the *d*_xz,yz_ forms a singlet with the organic ligand states, which reduces the effective spin seen by the substrate's quasiparticles to *S*=1 (ref. 30). The unpaired spin in the 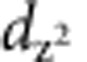 orbital is subject to strong coupling with the electronic states of the substrate leading to sizable Kondo screening, whereas the spin in the *d*_xy_ orbital is not expected to show a significant coupling with the substrate^24^,^30^,^31^. The occurrence of the Shiba states in tunnelling spectra is thus linked to the interaction of the 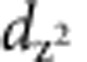 orbital with the substrate. We label this scattering channel as *k*=1 (sketches in Fig. 4). The unscreened spin in the *d*_xy_ orbital (which we label as *k*=2) does not give rise to an observable Shiba state in agreement with the theoretical predictions^30^. However, it couples to the spin in the 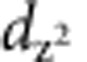 orbital and leads to an anisotropy splitting of the Shiba state with *k*=1 as discussed below.

We can describe the whole set of spectra on the different molecules by these spin states and their interactions: the coupling strength *J*_1_ of channel *k*=1 depends on the adsorption site of MnPc on the Pb(111) surface. In the case of strong coupling, the spin is totally Kondo screened (white arrow, *E*_b_<0, Fig. 4a) but *k*=2 remains unscreened (red arrow). Hence, the effective total spin is reduced to *S*=1/2 by Kondo screening. Tunnelling into the Shiba state reflects the excitation to the spin state S*=1. In the case of weak coupling in *k*=1 (left red arrow, *E*_b_>0; Fig. 4b), the total spin in the ground state multiplet is *S*=1. The excited state probed by the Shiba resonance is a S*=1/2 state, because the electron attached to *k*=1 must obey Pauli's exclusion principle and align anti-parallel.

Both spin-1 states, that is, S*=1 and *S*=1, can be split by magnetic anisotropy. A breaking of spherical symmetry of the Mn orbitals by the organic ligand and the adsorption on a substrate leads to a splitting of these spin states^22^,^23^. The corresponding Spin Hamiltonian 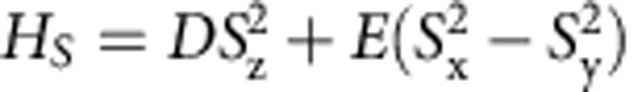, where the *S*_*i*_ are the spin operators in Cartesian coordinates, accounts for the axial anisotropy and additional rhombicity with the parameters *D* and *E*, respectively. This yields a new set of spin eigenstates 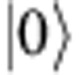, 
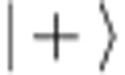
 and 
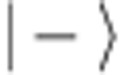
, with the latter two being linear combinations of the two former eigenstates with magnetic spin quantum numbers *m*_s_=1 and *m*_s_=−1, respectively^23^. Schemes of the excitations observed in the tunnelling spectra are shown in Fig. 4a,b. In the strongly coupled regime (Kondo screened), the excitation of the system yields three excited states with the energy splittings being related to the anisotropy parameters *D* and *E*. It should be noted that their values are not a direct measure of the anisotropy energies of the molecule on the surface, but rather represent a renormalized value due to the many-body interactions with the substrate^10^. As the energy separation between *E*_b1_ and *E*_b2_ is smaller than between *E*_b2_ and *E*_b3_, the anisotropy parameter *D* is negative, which means easy-axis anisotropy. For the weakly coupled system, that is, the free-spin case, the ground state is split by anisotropy. The excitation spectra reflect transitions from the states 
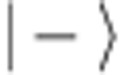
, 
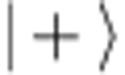
 and 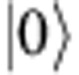 into the excited S*=1/2 state.

The characteristic variations of the peak areas are also well captured in this scenario of anisotropy-split Shiba states: all three spin excitations in the Kondo-screened regime are equally probable, therefore leading to the same relative peak area (Fig. 3c). On the other hand, a splitting of the ground state, as it is found in the free-spin regime, leads to a Boltzmann occupation of the levels. In the zero-temperature limit, which is discussed in ref. 10, only one excitation would be detected in the free-spin regime. At finite temperature, the levels are occupied according to Boltzmann statistics. The excitation probabilities are proportional to the state occupation and should thus directly reflect this distribution. Our data in Fig. 3d is in agreement with a Boltzmann distribution at 1.2 K or slightly higher, which reflects a temperature-induced population of the split ground state.

## Discussion

Our study shows the importance of anisotropy effects on the subgap excitations, which determine the electron transport properties. They provide an unambiguous fingerprint of the nature of the ground and excited state throughout the whole range of magnetic interaction strengths, which drive the phase transition between the two quantum ground states. Although the anisotropy energies are renormalized by the coupling to the substrates quasiparticles, this method can be used to extract knowledge about magnetic anisotropy and, hence, about spin–orbit coupling of magnetic adsorbates on superconductors.

Peculiar consequences of the split Shiba states may occur for coupled magnetic impurities, which lead to the formation of extended Shiba bands. If the exchange coupling strength is similar to or smaller than the anisotropy energy, the Shiba bands are expected to reflect the splitting of the individual Shiba states. In recent times, subgap bands have gained particular importance in the search of topological phases and Majorana states in ferromagnetic chains coupled to an *s*-wave superconductor^5^. If an odd number of spin-polarized bands crosses the Fermi level, Majorana end states can form in the presence of an induced (non-trivial) topological gap. Considering that the energy level splitting amounts to about one-third of the superconducting gap, one may expect that a split Shiba band structure affects the number of crossing bands and the topological gap width, which is also in the order of 100 μeV^32^.

## Methods

### Experimental parameters

The experiments were carried out in a commercial Specs JT-STM operating at a base temperature of 1.2 K and a base pressure below 10^−10^ mbar. The Pb(111) single crystal surface was cleaned by repeated cycles of Ne^+^ sputtering and annealing to 430 K until a clean, atomically flat and superconducting surface was obtained. From a Knudsen cell held at 673 K, MnPc was thermally evaporated onto the clean surface kept at room temperature, which then was precooled and transferred into the STM. To gain energy resolution beyond the Fermi–Dirac limit of a normal metal tip, we indented the chemically etched W tip into the clean, superconducting Pb(111) surface applying 100 V tip bias until a Pb-covered superconducting tip was obtained (energy resolution better than 45 μeV)^27^. The resulting spectrum as acquired on the clean Pb(111) surface is shown in Fig. 1b. We acquired *dI*/*dV* spectra as a function of sample bias under open-feedback conditions using conventional lock-in technique with a bias modulation of 15 μV_rms_ at an oscillation frequency of 912 Hz.

### Numerical deconvolution procedure and quality of the fit

A numerical deconvolution routine similar to ref. 27 has been used to extract the spectral intensity and binding energies *E*_b_ of the Shiba states from the *dI*/*dV* spectra. As all spectra are acquired in the weak tunnelling regime, which allows describing the tunnelling current in the single-electron approximation, we treat the spectral weight of the Shiba states as a density of states^14^. We calculate the tunnelling current using


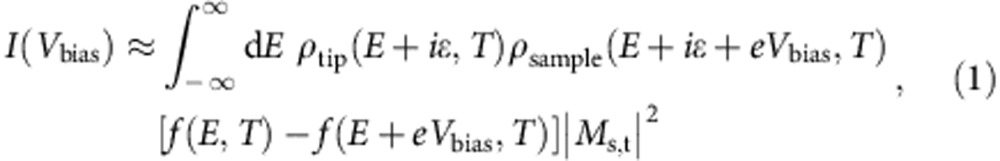


where *f*(*E*, *T*) is the Fermi–Dirac distribution, which depends on the energy *E* and the temperature *T*, *ρ*_tip_ (*ρ*_sample_) is the tip (sample) density of states and *iɛ* is the imaginary energy. We approximate the tunnel matrix element *M*_s,t_ as being constant, which is reasonable for the energy range of only a few meV.

We model the density of states of the superconducting tip *ρ*_tip_ according to the Bardeen–Cooper–Schrieffer (BCS) theory with the superconducting gap parameter Δ_tip_. Finite lifetime effects leading to a broadening of the coherence peaks are accounted for by an imaginary energy *iɛ* (refs 27, 33). We note the absence of the BCS peaks in the spectra on MnPc. This is due to the depletion of the BCS density of states by the formation of Bogoliubov-de Gennes quasiparticle excitations^28^. Therefore, the density of states of the substrate *ρ*_sample_ is modelled with symmetric step functions at ±Δ_sample_. The Shiba states are described by Lorentzian peaks at energies ±*E*_b*i*_. Their widths (Γ_*i*_) and areas (*A*_*i*_) are independent of each other. The density of states of the MnPc/Pb(111) surface thus reads:


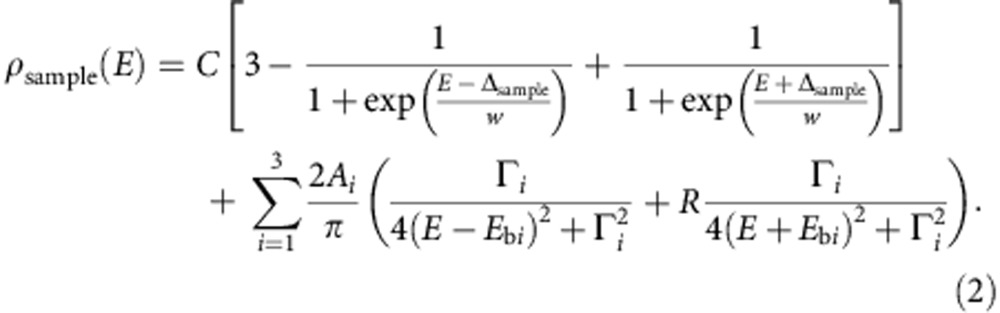


Here, *C* is the amplitude, which scales the step functions of width *w*. The ratio *R* accounts for the different heights of the Lorentzian peaks at positive and negative bias, respectively. A fit to the experimental data is obtained from the numerical derivative *dI*/*dV*.

In Supplementary Fig. 2, spectra of the three coupling regimes are shown together with the numerical fit. These spectra correspond to spectra I, II and III of Fig. 2. The fits reproduce the experimental spectra well besides some deviations of the height of the thermal resonances at 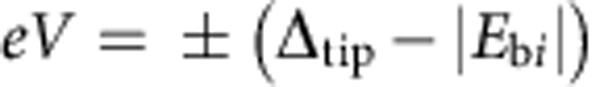 (spectrum III).

## Additional information

**How to cite this article:** Hatter, N. *et al.* Magnetic anisotropy in Shiba bound states across a quantum phase transition. *Nat. Commun.* 6:8988 doi: 10.1038/ncomms9988 (2015).

## Supplementary Material

Supplementary Information Supplementary Figures 1-2, Supplementary Note 1 and Supplementary References.

## References

[b1] Yu L. Bound state in superconductors with paramagnetic impurities. Acta Phys. Sin. 21, 75–91 (1965).

[b2] Shiba H. Classical spins in superconductors. Prog. Theor. Phys. 40, 435–451 (1968).

[b3] Rusinov A. I. On the theory of gapless superconductivity in alloys containing paramagnetic impurities. Zh. Eksp. Teor. Fiz. 56, 2047–2056 (1969) [Sov. Phys. JETP 29, 1101-1106 (1969)].

[b4] Pientka F., Glazman L. I. & von Oppen F. Topological superconducting phase in helical Shiba chains. Phys. Rev. B 88, 155420 (2013).

[b5] Nadj-Perge S. *et al.* Observation of Majorana fermions in ferromagnetic atomic chains on a superconductor. Science 346, 602–607 (2014). 25278507 10.1126/science.1259327

[b6] Röntynen J. & Ojanen T. Topological superconductivity and high chern numbers in 2D ferromagnetic shiba lattices. Phys. Rev. Lett. 114, 236803 (2015). 26196820 10.1103/PhysRevLett.114.236803

[b7] Ginsberg D. M. Consequences of Shiba's theory of magnetic impurities in superconductors, beyond s-wave scattering. Phys. Rev. B 20, 960–962 (1979).

[b8] Kunz A. B. & Ginsberg D. M. Band calculation of the effect of magnetic impurity atoms on the properties of superconductors. Phys. Rev. B 22, 3165–3172 (1980).

[b9] Flatté M. E. & Byers J. M. Local electronic structure of a single magnetic impurity in a superconductor. Phys. Rev. Lett. 78, 3761–3764 (1997).

[b10] Žitko R., Bodensiek O. & Pruschke T. Effects of magnetic anisotropy on the subgap excitations induced by quantum impurities in a superconducting host. Phys. Rev. B 83, 054512 (2011).

[b11] Golež D., Bonča J. & Žitko R. Vibrational Andreev bound states in magnetic molecules. Phys. Rev. B 86, 085142 (2012).

[b12] Kim Y., Zhang J., Rossi E. & Lutchyn R. M. Impurity-induced bound states in superconductors with spin-orbit coupling. Phys. Rev. Lett. 114, 2368040 (2015). 10.1103/PhysRevLett.114.236804 26196821

[b13] Ji S. H. *et al.* High-resolution scanning tunneling spectroscopy of magnetic impurity induced bound states in the superconducting gap of Pb thin films. Phys. Rev. Lett. 100, 226801 (2008). 18643441 10.1103/PhysRevLett.100.226801

[b14] Ruby M. *et al.* Tunneling processes into localized subgap states in superconductors. Phys. Rev. Lett. 115, 087001 (2015). 26340200 10.1103/PhysRevLett.115.087001

[b15] Matsuura T. The effects of impurities on superconductors with Kondo effect. Prog. Theor. Phys. 57, 1823–1835 (1977).

[b16] Franke K. J., Schulze G. & Pascual J. I. Competition of superconducting phenomena and Kondo screening at the nanoscale. Science 332, 940–944 (2011). 21596987 10.1126/science.1202204

[b17] Bauer J., Pascual J. I. & Franke K. J. Microscopic resolution of the interplay of Kondo screening and superconducting pairing: Mn-phthalocyanine molecules adsorbed on superconducting Pb(111). Phys. Rev. B 87, 075125 (2013).

[b18] Yazdani A., Jones B. A., Lutz C. P., Crommie M. F. & Eigler D. M. Probing the local effects of magnetic impurities on superconductivity. Science 275, 1767–1770 (1997). 9065395 10.1126/science.275.5307.1767

[b19] Balatsky A. V., Vekhter I. & Zhu J.-X. Impurity-induced states in conventional and unconventional superconductors. Rev. Mod. Phys. 78, 373–433 (2006).

[b20] Deacon R. S. *et al.* Tunneling spectroscopy of Andreev energy levels in a quantum dot coupled to a superconductor. Phys. Rev. Lett. 104, 076805 (2010). 20366905 10.1103/PhysRevLett.104.076805

[b21] Lee E. J. H. *et al.* Spin-resolved Andreev levels and parity crossings in hybrid superconductor-semiconductor nanostructures. Nat. Nanotechnol. 9, 79–84 (2014). 24336403 10.1038/nnano.2013.267

[b22] Gatteschi D., Sessoli R. & Villain J. Molecular Nanomagnets Oxford University Press (2006).

[b23] Tsukahara N. *et al.* Adsorption-induced switching of magnetic anisotropy in a single iron(II) phthalocyanine molecule on an oxidized Cu(110) surface. Phys. Rev. Lett. 102, 167203–167206 (2009). 19518750 10.1103/PhysRevLett.102.167203

[b24] Kügel J. *et al.* Relevance of hybridization and filling of 3d orbitals for the kondo effect in transition metal phthalocyanines. Nano Lett. 14, 3895–3902 (2014). 24871813 10.1021/nl501150k

[b25] Ji S. H. *et al.* Kondo effect in self-assembled manganese phthalocyanine monolayer on Pb islands. Chin. Phys. Lett. 27, 087202 (2010).

[b26] Stróżecka A., Soriano M., Pascual J. I. & Palacios J. J. Reversible change of the spin state in a manganese molecule. Phys. Rev. Lett. 109, 147202 (2012). 23083274 10.1103/PhysRevLett.109.147202

[b27] Ruby M., Heinrich B. W., Pascual J. I. & Franke K. J. Experimental demonstration of a two-band superconducting state for lead using scanning tunneling spectroscopy. Phys. Rev. Lett. 114, 157001 (2015). 25933331 10.1103/PhysRevLett.114.157001

[b28] Salkola M. I., Balatsky A. V. & Schrieffer J. R. Spectral properties of quasiparticle excitations induced by magnetic moments in superconductors. Phys. Rev. B 55, 12648–12661 (1997).

[b29] Liao M.-S., Watts J. D. & Huang M.-J. DFT study of unligated and ligated manganes^II^ porphyrins and phthalocyanines. Inorg. Chem. 44, 1941–1949 (2005). 15762720 10.1021/ic0401039

[b30] Jacob D., Soriano M. & Palacios J. J. Kondo effect and spin quenching in high-spin molecules on metal substrates. Phys. Rev. B 88, 134417 (2013).

[b31] Kügel J. *et al.* State identification and tunable Kondo effect of MnPc on Ag(001). Phys. Rev. B 91, 235130 (2015).

[b32] Ruby M., Pientka F., Peng Y., von Oppen F., Heinrich B. W. & Franke K. J. End states and subgap structure in proximity-coupled chains of magnetic adatoms. Preprint at http://arxiv.org/abs/1507.03104 (2015). 10.1103/PhysRevLett.115.197204 26588411

[b33] Dynes R. C., Narayanamurti V. & Garno J. P. Direct measurement of quasiparticle-lifetime broadening in a strong-coupled superconductor. Phys. Rev. Lett. 41, 1509–1512 (1978).

