# Supplementary material for: Magnetic anisotropy in Shiba bound states across a quantum phase transition
Source: Nat Commun. 2015 Nov 25;6:8988. doi: 10.1038/ncomms9988 (PMC4674822; doi:10.1038/ncomms9988)
Supplement: Supplementary Information — Supplementary Figures 1-2, Supplementary Note 1 and Supplementary References. [file ncomms9988-s1.pdf]

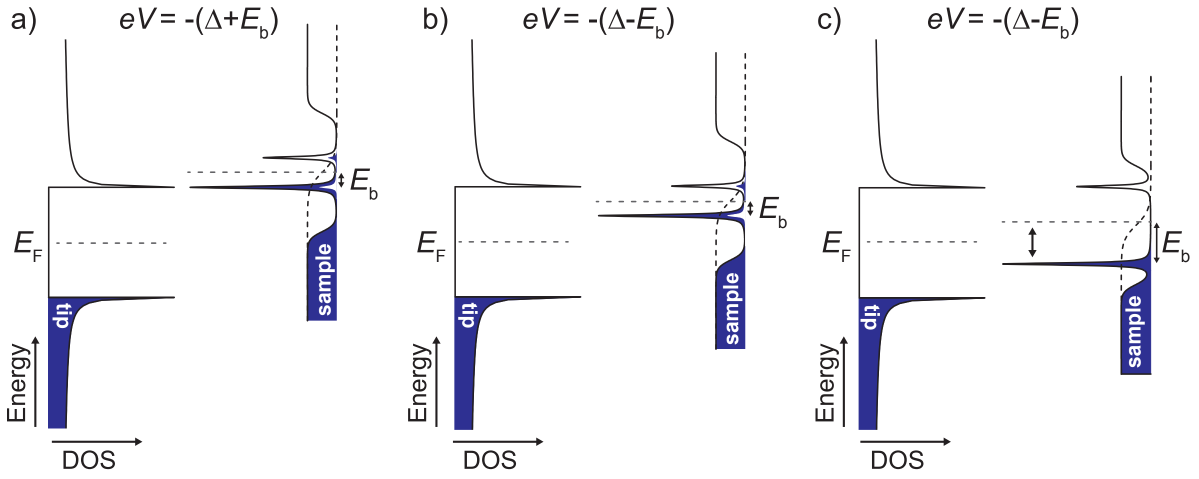

Supplementary Figure 1. Sketch of tunneling processes at negative bias voltage: (a) At $eV = -(\Delta + E_\mathrm{b})$ the alignment of an occupied Shiba state (binding energy E_b) in the sample density of states (DOS) with the unoccupied Bardeen-Cooper-Schrieffer (BCS) peak in the tip DOS marks the onset of tunneling. (b) For finite temperatures and small binding energies E_b, thermal occupation of the Shiba state yields tunneling also at $eV = -(\Delta - E_\mathrm{b})$. (c) For larger binding energies E_b, thermally assisted tunneling at $eV = -(\Delta - E_\mathrm{b})$ vanishes. The dashed line shows the occupation probability, which follows the Fermi-Dirac distribution function. The resulting occupation/depletion of states is illustrated by shaded/emptied areas in the peaks.

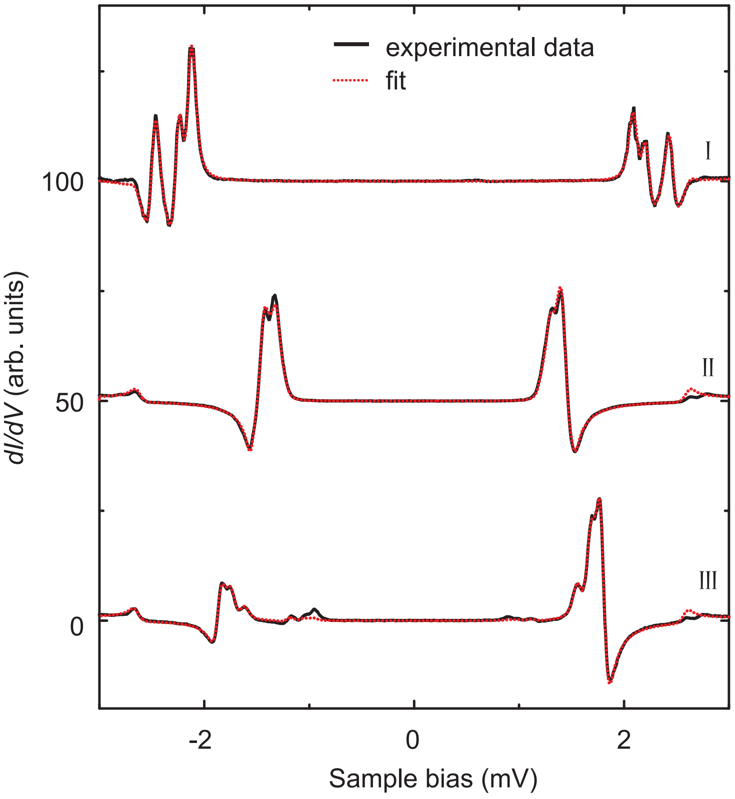

Supplementary Figure 2. Demonstration of the fit quality: spectra I, II, and III of Figure 2 of the main manuscript are superimposed with their respective fits. The fit procedure is described in the Methods section of the manuscript.

SUPPLEMENTARY NOTE 1:

Tunneling processes between Shiba bound states and the superconducting tip

All differential conductance spectra acquired with a superconducting tip on the Manganese phthalocyanine (MnPc) molecules on Pb(111) show one triplet of peaks in the bias voltage window $-2\Delta/e < V_{\mathrm{bias}} < -\Delta/e$ and one in $\Delta/e < V_{\mathrm{bias}} < 2\Delta/e$. If the triplets are located deep inside the gap, additional resonances appear in the energy interval $[-\Delta_{\mathrm{tip}}, \Delta_{\mathrm{tip}}]$. All spectra are acquired in the weak tunneling regime, which allows describing the tunneling current as single-electron tunneling processes between the density of states of tip and substrate [1].

In Supplementary Figure 1 we plot the schemes of tunneling between a superconducting tip and a single Shiba state at E_{b}. Note that these tunneling processes can be easily generalized to multiple Shiba resonances. At $eV = -(\Delta + E_{\mathrm{b}})$ the tip's unoccupied Bardeen-Cooper-Schrieffer (BCS) resonance is aligned with the occupied Shiba state of the substrate. This marks the onset of single electron tunneling from the substrate to the tip with increasing bias voltage. The reverse tunneling process from the occupied states in the tip to the unoccupied Shiba state of the substrate sets in at the threshold of $eV = \Delta + E_{\mathrm{b}}$. A finite temperature leads to a thermal occupation of the Shiba states. The occupation probability follows the Fermi-Dirac distribution function sketched as a dashed line in Supplementary Figure 1. The resulting occupation/depletion of states (marked by the shaded/emptied areas in the peaks) enables a finite tunneling current at the threshold of $eV = \pm(\Delta - E_{\mathrm{b}})$. When the Shiba states are sufficiently deep inside the gap, this occupation is non-negligible and gives rise to the peaks within $[-\Delta_{\mathrm{tip}}, \Delta_{\mathrm{tip}}]$ [Supplementary Figure 1(b)]. The further away the Shiba state is from E_{F}, the smaller is the thermal occupation, which eventually completely suppresses the tunneling [Supplementary Figure 1(c)].

SUPPLEMENTARY REFERENCES

[1] Ruby, M., Pientka, F., Peng, Y., von Oppen, F., Heinrich, B. W. & Franke, K. J. Tunneling processes into localized subgap states in superconductors. *Phys. Rev. Lett.* **115**, 087001 (2015).
